# Benign mesenteric lymphangioma presenting as acute pancreatitis: a case report

**DOI:** 10.1186/1757-1626-2-9328

**Published:** 2009-12-16

**Authors:** Solomon Akwei, Neil Bhardwaj, Paul D Murphy

**Affiliations:** 1 Department of Surgery, Queens Medical Centre, Derby Road, Nottingham, NG7 2UH, UK; 2 Department of Surgery, Leicester General Hospital, Gwendolen Road, Leicester, LE5 4PW, UK; 3 Department of Surgery, Pilgrim Hospital, Sibsey Road, Boston, Lincolnshire, PE21 9QS, UK

## Abstract

Benign mesenteric lymphangiomas are rare intra-abdominal cysts which may be asymptomatic or present with a variety of abdominal symptoms including an acute abdomen. We are however not aware of any reports in the literature linking mesenteric lymphangioma to acute pancreatitis. We present the case of a 62-year-old man who was admitted with signs and symptoms of acute pancreatitis and a palpable abdominal mass. Computerised tomography (CT) of his abdomen confirmed the presence of a mesenteric cystic mass. He underwent a laparotomy at which a large thin walled mass filled with a chylous fluid was resected. Histological analysis of this cyst showed it to be a benign mesenteric lymphangioma.

## Case presentation

**Figure 1:**
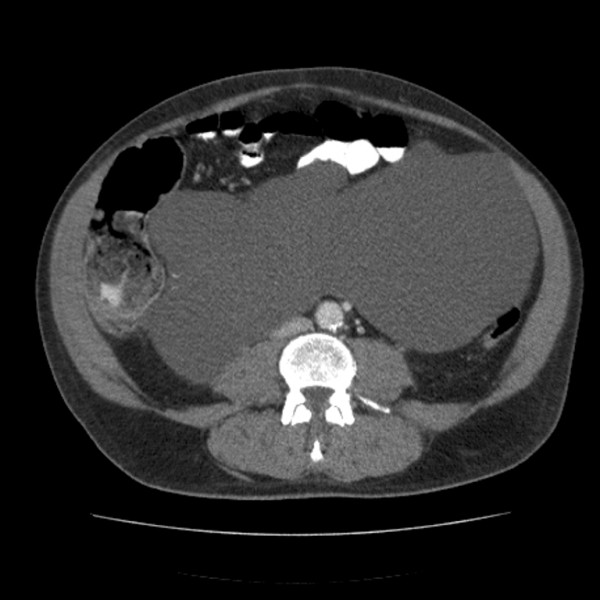
**CT scan performed on initial admission showing large intra-abdominal cystic lesion**.

A 62-year-old Caucasian man, without a previous history of pancreatitis, gallstones or heavy alcohol consumption was admitted with 36 hr history of acute generalised abdominal pain associated with nausea and multiple episodes of vomiting. Other than a previous history of an inguinal hernia repair and a hiatus hernia, his past medical history was unremarkable. His only regular medication was a proton-pump inhibitor. Clinical examination revealed he was pyrexial (Temperature 38.2°C) and had a soft, distended, abdomen with palpable diffuse soft mass in the central abdominal area which was moderately tender. His chest X-ray was unremarkable, his abdominal X-ray in hindsight shows some peripheral displacement of small bowel, but was otherwise also unremarkable. His blood biochemistry showed an elevated serum amylase (762 u/dL) and an elevated alanine aminotransferase 191 IU/L (3-35). Other liver function test results were, total bilirubin 24 u/L (3 - 25), alkaline phosphatise 139 u/L (38 - 149) Gamma-glutamyl transferase was also elevated at 508 u/l (12-64). Serum lipase was not checked. His haemoglobin was 14.5 g/dl and white cell count was 13 × 10^3^/μL. A working diagnosis of acute pancreatitis and a palpable mass of unknown significance was made. CT scan of the abdomen revealed a large multi-lobulated cyst within the small bowel mesentery (Figure 1). There was also radiological evidence of peripancreatic inflammation with a normally enhancing pancreas. Abdominal ultrasonography showed a normal appearances of the liver, gallbladder and bile ducts, and no calculi. It also showed an anechoic septated mass which was indistinguishable from the pancreas. His symptoms resolved over the next 48 hrs with conservative measures. He was discharged with a plan for elective laparotomy and excision of what was at the time a non-specific intra-abdominal cystic mass.

**Figure 2:**
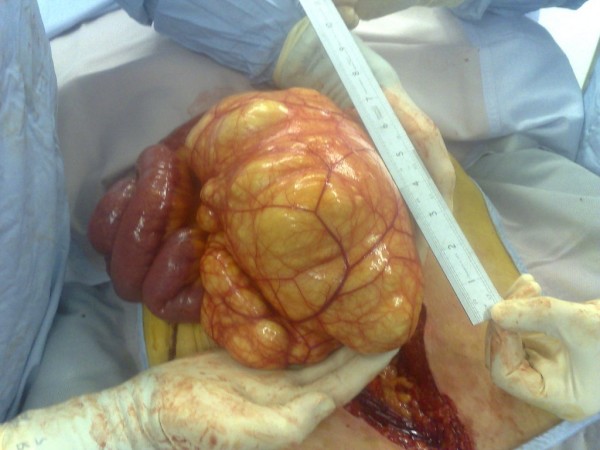
**Intra-operative appearance of the thin walled lymphangioma containing chylous fluid**.

**Figure 3:**
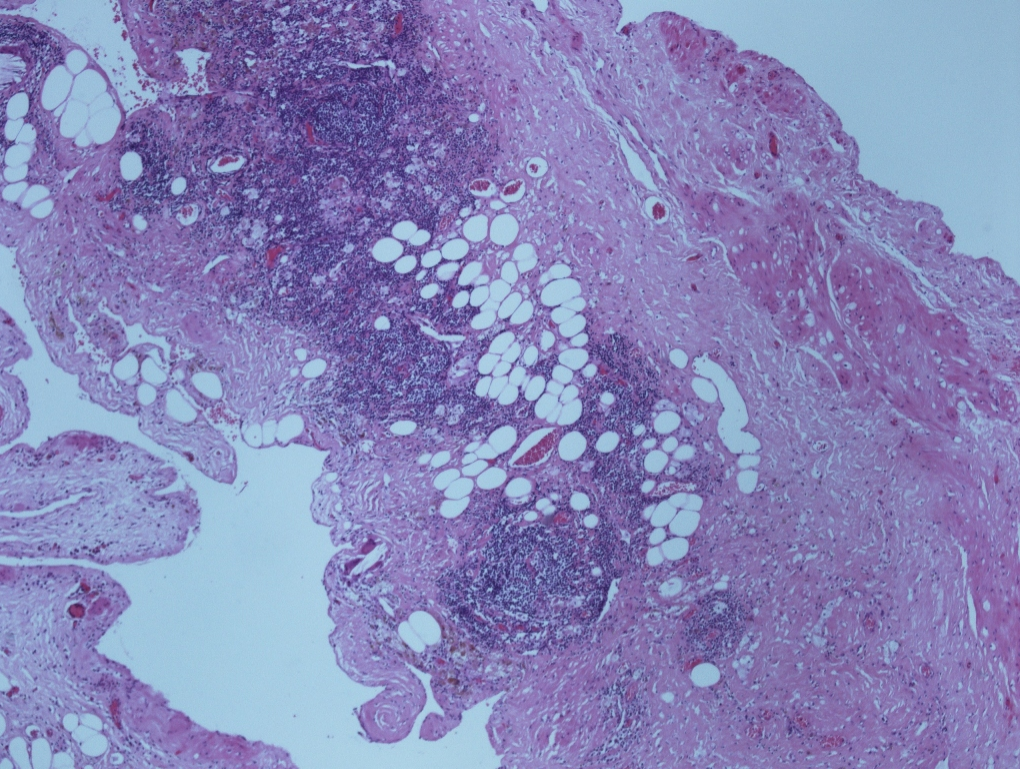
**H&E section ×40 magnification - shows wall between two locules containing fibrous tissue, fat, smooth muscle bundles and small collections of lymphocytes**.

At laparotomy, a large thin walled multi-loculated mass measuring 25 × 15 × 10 cm (Figure 2) was found within the proximal small bowel mesentery, a part of which was adherent to the head of the pancreas. The pancreas was otherwise normal in appearance, so were the liver and gallbladder. A small part of the mass adherent to the pancreas could not be fully resected without causing damage to the pancreas; and hence was left in situ. The mass contained approximately 3 litres of a chylous fluid, biochemical analysis of which showed elevated levels of cholesterol (7.9 mmol/L) and triglyceride (18.4 mmol/L) but no evidence of pancreatic fluid. Histological analysis of the resected specimen showed a complex multiloculated lesion with locules lined by flattened cells and intermittent smooth muscle bundles. It also had small vascular channels associated with lymphoid aggregates (Figure 3). These findings were consistent with a diagnosis of a benign mesenteric lymphangioma.

In the early post-operative period, he made good progress but began showing signs of infection, with rising infection markers, pyrexia and abdominal discomfort. A CT scan of the abdomen revealed a pelvic collection requiring a CT-guided drain. This collection was purulent in appearance, and *Escherichia coli *was cultured from it. He settled symptomatically with drainage of the collection and antibiotic treatment, and was subsequently discharged three weeks later. He represented one month after discharge with a recurrent intra-abdominal collection which also necessitated CT guided drain. There was no further recurrence of a collection after this. No recurrence of the cyst has been noted after a year of follow-up.

## Discussion

A diagnosis of acute pancreatitis should be considered in a patient presenting with acute abdominal pain, nausea and vomiting. The cause of pancreatitis is diverse but is often associated with gallstone disease or excess alcohol and can be discerned from the history and initial laboratory investigations. Other more obscure causes require further investigation. Approximately 10% are said to have idiopathic pancreatitis, where no obvious case is found [1].

Our patient presented with typical symptoms of acute pancreatitis. He had evidence of peripancreatic inflammation on his CT scan and elevated serum amylase. Abdominal ultrasonography and CT also did not show demonstrate gallstones. Other liver function tests were not consistent with gallstone disease and he did not have a history of alcohol excess. Intra-operatively, the mass was found to be adherent to the head of the pancreas.

Benign mesenteric lymphangiomas are rare intra-abdominal cysts thought to account for between 1 in 100,000 to 1 in 250,000 of hospital admissions [2,3]. Lymphangiomas in general are commoner in children, 40% usually present by age 1 and 80% by age 5 [3]. It is thus unusual for lymphangioma to be seen in an older adult as in our case. Lymphangiomas can occur throughout the body but commonest site is usually the neck, where they are referred to as cystic hygromas. Within the abdomen, the commonest site is the mesentery [4,5] but may also occur in the omentum, retroperitoneum and mesocolon [5,6]. Lymphangiomas of the pancreas has also been reported [7].

The aetiology of lymphangiomas remains unclear. Congenital developmental defects of the lymphatics have been proposed, such as the proliferation and dilatation of blind ended lymphatic sacs which lack proper connections with the venous system [4]. The theory of a congenital aetiology may be supported by the fact that most cases present in childhood. Other potential causes are thought to include, abdominal trauma, localised lymphatic degeneration and lymphatic obstruction [8,9].

Clinically, intra-abdominal lymphangiomas may be asymptomatic; however a gradual increase in abdominal girth, sensation of fullness and vague abdominal pain should alert the clinician of this rare condition. Pain may range from chronic background abdominal pain to more acute pain due to torsion or haemorrhage into the cyst, compression of surrounding structures or intestinal obstruction. They can also infiltrate into surrounding viscera and lead to organ dysfunction [8]. Although cystic lymphangiomas of the pancreas have been reported [8], we are not aware of any reports of mesenteric lymphangioma presenting as acute pancreatitis as in our case. It is likely that the part of the cyst found to be adherent to the pancreas caused pancreatitis through compression or local irritation.

The diagnosis of mesenteric lymphangioma is usually made histologically following surgical resection of the cyst. There are no blood tests which will confirm the diagnosis. Radiological investigations using ultrasound or computerised tomography (CT) usually confirms the presence of a mass and may help to exclude other causes of intra-abdominal masses but is usually insufficient to provide a definite diagnosis. MRI has also been proposed as a suitable investigation for defining the origins of the mass and thus aid diagnosis [8]. However, Jain et al report using multislice spiral CT to confidently establish a diagnosis prior to surgery [10].

Treatment of mesenteric cysts is primarily by surgical resection. This has traditionally been through open surgery, however laparoscopic resection has been reported [8]. Occasionally resection of adjacent structures such as small bowel may also be required where the cyst intimately involves the bowel or important vessels. In our case the cyst wall was adherent to the pancreas, thus making complete resection impossible without damage to the pancreas. It is likely that the remnant of the cyst wall may have contributed to the recurrent collections our patient developed following his surgery. The use of alcohol [5] and OK432 [8] have also been reported as an alternative is cases where surgical resection is not a viable option.

Our case illustrates an unusual presentation of benign lymphangioma and options for treatment. Although rare, clinicians should consider mesenteric lymphangioma in the differential diagnoses of patients found to have intra-abdominal cystic masses on abdominal CT scans.

## Competing interests

The authors declare that they have no competing interests.

## Consent

Written informed consent was obtained from the patient for publication of this case report and accompanying images. A copy of the written consent is available for review by the Editor-in-Chief of this journal.

## Authors' contributions

SA did literature review and writing of manuscript. NB involved in literature review and contributed to writing and amending the manuscript. PDM supervised writing of the manuscript. All authors were also involved in the care of the patient and have approved the manuscript.
